# Synthesis of acetic acid via methanol hydrocarboxylation with CO_2_ and H_2_

**DOI:** 10.1038/ncomms11481

**Published:** 2016-05-11

**Authors:** Qingli Qian, Jingjing Zhang, Meng Cui, Buxing Han

**Affiliations:** 1 Beijing National Laboratory for Molecular Sciences, CAS Key Laboratory of Colloid, Interface and Chemical Thermodynamics, Institute of Chemistry, Chinese Academy of Sciences, University of Chinese Academy of Sciences, Beijing 100190, China

## Abstract

Acetic acid is an important bulk chemical that is currently produced via methanol carbonylation using fossil based CO. Synthesis of acetic acid from the renewable and cheap CO_2_ is of great importance, but state of the art routes encounter difficulties, especially in reaction selectivity and activity. Here we report a route to produce acetic acid from CO_2_, methanol and H_2_. The reaction can be efficiently catalysed by Ru–Rh bimetallic catalyst using imidazole as the ligand and LiI as the promoter in 1,3-dimethyl-2-imidazolidinone (DMI) solvent. It is confirmed that methanol is hydrocarboxylated into acetic acid by CO_2_ and H_2_, which accounts for the outstanding reaction results. The reaction mechanism is proposed based on the control experiments. The strategy opens a new way for acetic acid production and CO_2_ transformation, and represents a significant progress in synthetic chemistry.

## Abstract (web summary)

Industrial routes to acetic acid use carbon monoxide for the carbonylation of methanol. Here, the authors report a hydrocarboxylation method that instead uses carbon dioxide and hydrogen for the conversion of methanol into acetic acid.

## Graphical abstract

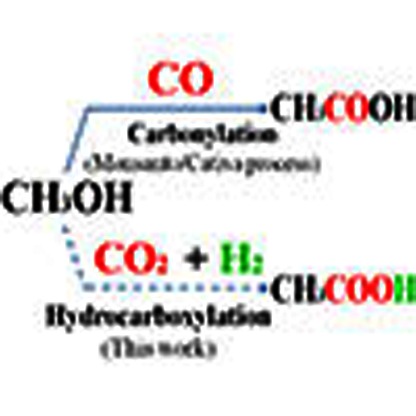

Acetic acid is an important bulk chemical^1^,^2^ that is currently produced via methanol carbonylation (CH_3_OH+CO→CH_3_COOH), such as Monsanto process^3^. CO_2_ is a greenhouse gas^4^ and its fixation into value-added chemicals is highly desirable for a sustainable society^5^. So far, CO_2_ has been utilized to synthesize various chemicals^6^,^7^,^8^,^9^,^10^,^11^, such as alcohols, urea, carbonates, polymers and carboxylic acids. In the field of carboxylic acids syntheses using CO_2_, major advance has been focused on hydrogenating CO_2_ into formic acid or its derivatives^12^,^13^,^14^,^15^,^16^,^17^,^18^,^19^ and hydrocarboxylating unsaturated hydrocarbons or nucleophiles into fine chemicals^20^,^21^,^22^,^23^,^24^. Synthesis of acetic acid utilizing CO_2_ is of great importance, but is challenging. The reported routes suffer from obvious disadvantages, such as low selectivity, low activity, higher reaction temperature and use of expensive and/or toxic reactants^25^,^26^,^27^,^28^,^29^. For example, acetic acid could form slowly with low selectivity when CO_2_ was reduced by iron nanoparticles^25^. Synthesis of acetic acid from CO_2_ and CH_4_ is thermodynamically unfavourable, thus high temperature is required and the acetic acid yield is very low^26^,^27^. Trace acetic acid in CO_2_ hydrogenation was detected where CO accounted for 96% of the total product^28^. When methyl iodide (CH_3_I), CO_2_ and H_2_ were used as reactants acetic acid was formed at low rate and selectivity (acetic acid 10.7%, CO 58.4%, and CH_4_ 30.9%)^29^. In addition, the reactant CH_3_I is toxic and expensive.

Here we show a protocol to produce acetic acid from CO_2_, methanol and H_2_ (Fig. 1). The reaction could proceed very efficiently by homogeneous catalysis under mild condition. Interestingly, the CO_2_ (not via CO) participates in acetic acid formation with H_2_, accounting for the outstanding reaction results. The strategy represents a significant progress in synthetic chemistry. Because the reported routes of hydrocarboxylation use other substrates, such as alkenes, alkynes, arenes and/or organic halides, and the metallic reducing agents are generally utilized^20^,^21^,^22^,^23^,^24^. This work opens a practical way to fix CO_2_ into bulk chemicals using easily available and cheap feedstocks, which is a promising countermeasure for mankind to solve the ever-increasing crisis in environment and resources.

## Results

### Catalytic system for acetic acid synthesis

The target reaction was catalysed effectively by Ru–Rh bimetallic catalyst using imidazole as the ligand and LiI as the promoter in 1,3-dimethyl-2-imidazolidinone (DMI) at milder conditions (Table 1). Acetic acid was the predominant product and other products being negligible in the reaction solution (Supplementary Fig. 1a). The turnover frequency (TOF) of acetic acid reached 30.8 h^−1^ and the yield of acetic acid based on methanol was 70.3% (Entry 1). The rest of the methanol was converted into CH_4_. Very interestingly, the CO was hardly detectable in the gaseous sample (Supplementary Fig. 1b).

The ligand was crucial to the catalytic performance. Without ligand, the catalyst was unstable with much lower activity and selectivity (Entry 2). We also tried other ligands, but the results were not satisfactory (Entries 3–6). So imidazole was the suitable ligand for the reaction. The high efficiency of imidazole in this reaction should be due to its good coordination capability with the active center, which will be discussed in detail in the following paragraph. The promoter was also indispensable in this reaction. Without promoter, no acetic acid was formed and the catalyst was unstable (Entry 7). When the promoters with other cations (Na^+^, K^+^ and Sn^4+^) or anions (Cl^−^ and Br^−^) were utilized, the results were poor (Entries 8–13). Therefore, LiI was the best promoter in catalysing the target reaction. The better performance of lithium cation may be due to its stronger Lewis acidity and proper ion size, which could render appropriate coordination sites during the reaction. The superiority of the iodide anion could be attributed to its stronger nucleophilicity, which would facilitate the C–C bond formation in the generation of acetic acid.

We tested Ru_3_(CO)_12_ as single catalyst but no acetic acid formed (Entry 14). When we tried Rh_2_(OAc)_4_ separately, acetic acid formed at a lower rate (Entry 15). Thus, Rh complex was the major catalyst and Ru complex was the co-catalyst. We have combined Rh_2_(OAc)_4_ with other Ru compounds, such as RuO_2_ or Ru(PPh_3_)_3_Cl_2_, but the reaction results were poor (Entries 16, 17). We also combined Ru_3_(CO)_12_ with other Rh compounds, such as RhCl_3_·3H_2_O or Rh(CO)H_2_(PPh_3_)_3_, but the efficiencies were also not satisfactory (Entries 18, 19). Obviously, synergistic effect existed between the Ru–Rh catalysts in accelerating the reaction (Entry 1). The superiority of the Ru_3_(CO)_12_/Rh_2_(OAc)_4_ in producing acetic acid could be ascribed to their fitness in triggering the synergistic effect.

The solvent effect is also important for the reaction. On the basis of Ru_3_(CO)_12_/Rh_2_(OAc)_4_, imidazole and LiI, other solvents were tested, but the catalytic performances were poor (Entries 20–24). When other solvents, such as DMF, tetrahydrofuran, cyclohexane and water, were used, the metal complex decomposed during the reaction and evident black precipitates were observed. The results indicate that DMI could stabilize the catalyst. As a weak Lewis base, DMI may also help to absorb and activate acidic CO_2_. Moreover, the DMI is stable under H_2_ atmosphere and the generation of acetic acid from acetate in Rh_2_(OAc)_4_ was excluded because the reaction did not occur when only H_2_ was used as the reactant (Entry 25). Hence, the catalytic system consisting of Ru_3_(CO)_12_, Rh_2_(OAc)_4_, imidazole, LiI, and DMI was the best for the target reaction.

### Effect of reaction parameters

**Figure 2:**
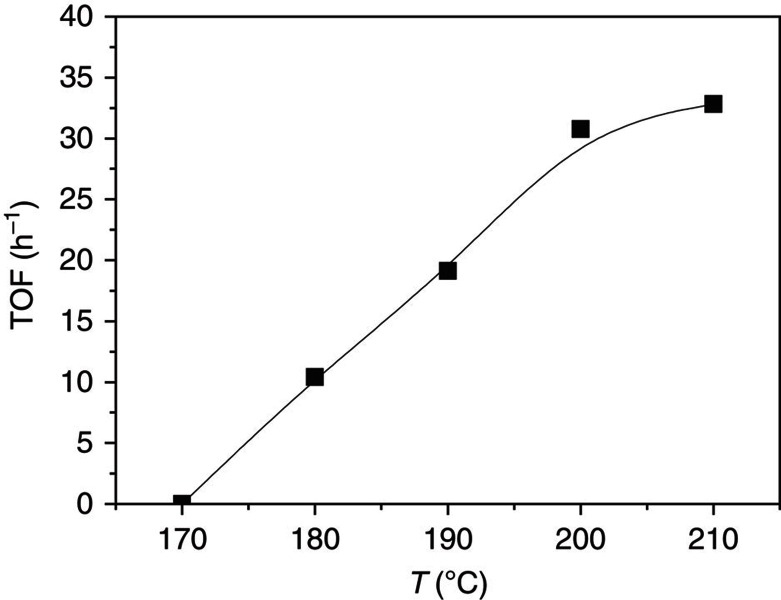
The TOF of acetic acid at different temperatures. Condition: 40 μmol Ru_3_(CO)_12_ and 40 μmol Rh_2_(OAc)_4_ (based on metals), 0.75 mmol imidazole, 3 mmol LiI, 2 ml DMI, 12 mmol MeOH, 4 MPa CO_2_ and 4 MPa H_2_ (at room temperature), and 12 h. TOF denotes moles of acetic acid produced per mole of Rh catalyst per hour in the steady state.

On the basis of the optimized catalytic system, we studied the effects of reaction temperature, pressure and dosage of each catalyst component on the reaction. Figure 2 shows the TOF of acetic acid at different temperatures. The acetic acid was not detectable when the reaction was carried out at 170 °C, and it emerged with remarkable amount when the temperature was elevated to 180 °C. The activity grew steadily with the increase of temperature until 200 °C. The TOF of acetic acid at 200 °C reached 30.8 h^−1^ and increased slowly when the temperature was further increased.

The results in Fig. 2 suggest that 200 °C is a suitable temperature. We further studied the effects of other parameters on the reaction at this temperature, and the results are given in Table 2. The pressure of the reaction gases (CO_2_ and H_2_) evidently affected the reaction. At fixed ratio of CO_2_ and H_2_ (1:1), the yield of acetic acid increased markedly as the total pressure was raised from 2 to 10 MPa (Entries 1–5). At a fixed total pressure of 8 MPa, the ratio of CO_2_ and H_2_ also influenced the reaction and highest yield of acetic acid was obtained at the ratio of 1:1 (Entries 4, 6, 7). In the absence of CO_2_ or H_2_, the reaction did not occur (Entries 8, 9). Hence both CO_2_ and H_2_ are necessary for the formation of acetic acid. These results demonstrated that acetic acid was not generated from the CO in Ru_3_(CO)_12_ via methanol carbonylation, and DMI was stable at the reaction condition.

The dosages of imidazole and LiI also influenced the reaction significantly. The yield of acetic acid was the highest when 750 μmol of imidazole was used (Entries 4, 10, 11), and the highest yield occurred at LiI dosage of 3 mmol (Entries 4, 12, 13). The results indicate that excess amount of imidazole or LiI was not favourable to the reaction. The main reason may be that the active sites were occupied by the excess imidazole or iodide anions due to their good coordination capability, and the reaction was inhibited accordingly. The atom ratio of the Ru and Rh also affected the yield of the reaction. At the same total amount of Ru and Rh (80 μmol), 40 μmol Ru+40 μmol Rh gave the highest yield of acetic acid (Entries 4, 14, 15). As expected, the total yield of acetic acid increased with increasing catalyst dosage (Entries 4, 16, 17), but it was less sensitive when the amount of catalyst was large enough. The above results reveal that the reaction condition in Entry 1 of Table 1 was the optimal.

### Recyclability

**Figure 3:**
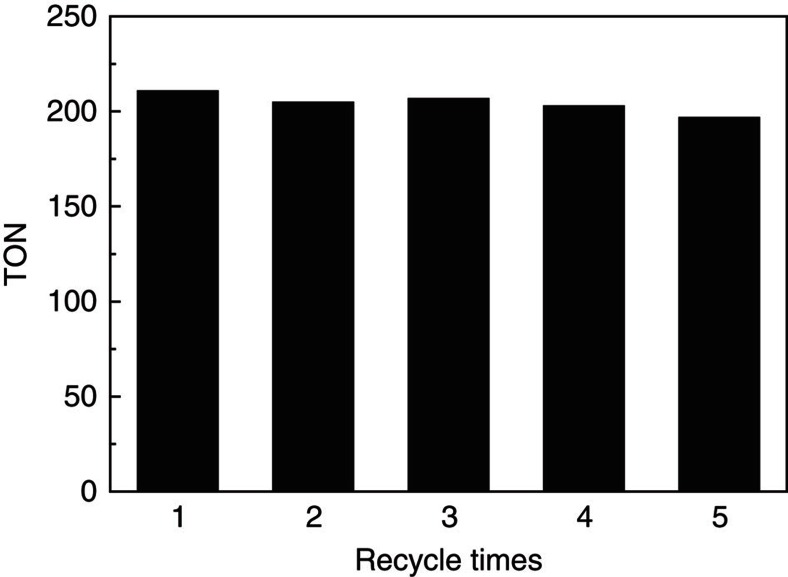
The results of the recycling test. Condition: 40 μmol Ru_3_(CO)_12_ and 40 μmol Rh_2_(OAc)_4_ (based on metals), 0.75 mmol imidazole, 3 mmol LiI, 2 ml DMI, 12 mmol MeOH, 4 MPa CO_2_ and 4 MPa H_2_ (at room temperature), 200 °C, and 12 h. TON denotes moles of acetic acid produced per mole of Rh catalyst.

To study the reusability of the catalytic system, the acetic acid generated in the reaction system was removed in a vacuum oven at 85 °C for 5 h, and GC analysis showed that the acetic acid remained in the reactor was negligible after the evacuation process, then the catalytic system was used directly for the next run. The results indicated that the catalytic activity did not change considerably after five cycles and the TON of acetic acid reached 1,022 in the five cycles. (Fig. 3).

### Time course of the reaction

**Figure 4:**
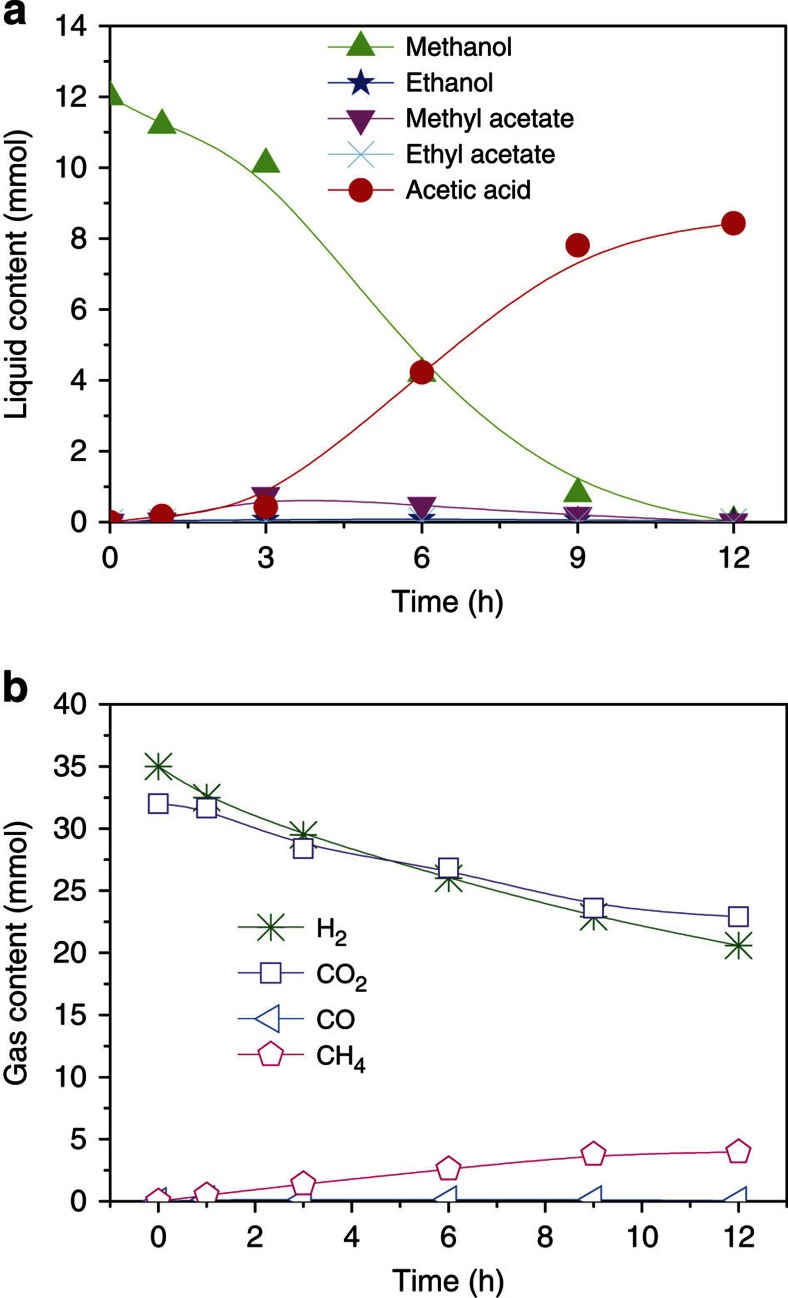
Time course of the methanol hydrocarboxylation. (**a**) Liquid content, (**b**) gas content. Condition: 40 μmol Ru_3_(CO)_12_ and 40 μmol Rh_2_(OAc)_4_ (based on metals), 0.75 mmol imidazole, 3 mmol LiI, 2 ml DMI, 12 mmol MeOH, 4 MPa CO_2_ and 4 MPa H_2_ (at room temperature), 200 °C.

Figure 4 presents the time course of the reaction. The amount of acetic acid increased slowly at the beginning (0–3 h) mainly because the acetic acid reacted with methanol to form methyl acetate. After that time the amount of acetic acid increased steadily (3–9 h). The reaction slowed down when methanol feedstock was gradually used up (9–12 h). As expected, with consumption of methanol, the methyl acetate formed initially was converted into acetic acid because of reverse esterification. The CO_2_ consumption directly correlated with the production of acetic acid. The amount of CH_4_ generated in the reaction was minor. Surprisingly, in the whole process, CO was nearly undetectable and alcohols formation was negligible.

### Role of the imidazole

To understand the above results, we studied the hydrogenation of CO using the catalytic system. The results showed that plenty of alcohols and CH_4_ were generated, and imidazole had no obvious impact on the reaction (Supplementary Figs 2 and 3). When we tried CO_2_ hydrogenation without imidazole, considerable amounts of CO, alcohols and CH_4_ were formed in the reaction (Supplementary Fig. 4). Because CO is a well-known intermediate in CO_2_ hydrogenation to generate alcohols and alkanes^30^, we could deduce that in the absence of imidazole the CO_2_ was firstly transformed into CO, then alcohols and CH_4_ were produced via CO hydrogenation. However, CO and liquid product formed were negligible when the imidazole was used in the CO_2_ hydrogenation reaction (Supplementary Fig. 5). Hence, we conclude that the imidazole inhibited hydrogenation of CO_2_ into CO, which is the origin for the excellent selectivity of acetic acid in this work. As we have mentioned in the former paragraph, the imidazole also played a key role in catalytic activity and stability. The X-ray photoelectron spectroscopy study revealed the facile coordination of imidazole with the Ru and Rh catalysts, which accounted for the role of imidazole in the reaction (Supplementary Fig. 6).

### Reaction pathway

**Figure 5:**
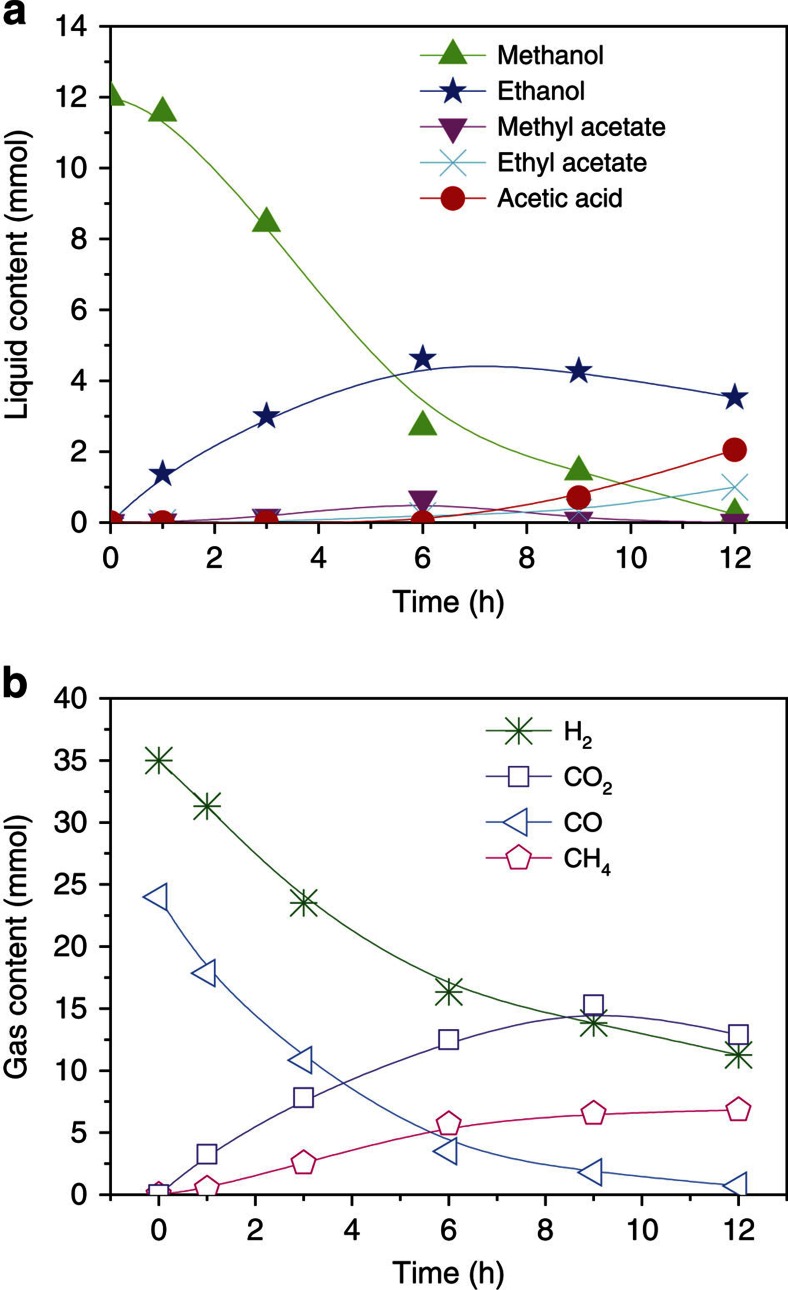
Time course of the reaction of methanol with CO and H_2_. (**a**) Liquid content, (**b**) gas content. Condition: 40 μmol Ru_3_(CO)_12_ and 40 μmol Rh_2_(OAc)_4_ (based on metals), 0.75 mmol imidazole, 3 mmol LiI, 2 ml DMI, 12 mmol MeOH, 4 MPa CO and 4 MPa H_2_ (at room temperature), 200 °C.

Production of acetic acid from CO and methanol, that is, methanol carbonylation, is a well-known reaction^3^. So there are two possible pathways of acetic acid synthesis from CO_2_, methanol and H_2_. The first is the CO pathway, that is, the CO_2_ was hydrogenated to CO, then acetic acid was formed by methanol carbonylation. The second is the CO_2_ pathway, namely, the methanol was hydrocarboxylated into acetic acid by CO_2_ and H_2_. All the above experimental results support the second pathway. To get further evidence to support the above argument, we studied the time course of the reaction of CO, methanol and H_2_ (Fig. 5). At the beginning, methanol was mostly homologated into ethanol by CO and H_2_, accompanying with gradual accumulation of CO_2_ because CO_2_ is a common byproduct in the methanol homologation, especially in the presence of amines^31^. At 6 h, CO in the reactor decreased to 3.5 mmol and CO_2_ increased to 12.5 mmol accordingly. At this point, the ethanol generation ceased and minor acetates formed. With time going on, the CO_2_ played a key role in the reaction. After 9 h, the CO_2_ content dropped obviously and considerable acetic acid and acetates emerged accordingly. These results rule out the possibility of first pathway (via CO). The CO_2_ pathway was further confirmed by tracer experiments using CH_3_OD, CH_3_^18^OH and ^13^CH_3_OH, respectively (Supplementary Figs 7, 8 and 9). To our knowledge, this is the first work on methanol hydrocarboxylation with CO_2_ and H_2_. It is an important contribution to synthetic chemistry.

## Discussion

**Figure 6:**
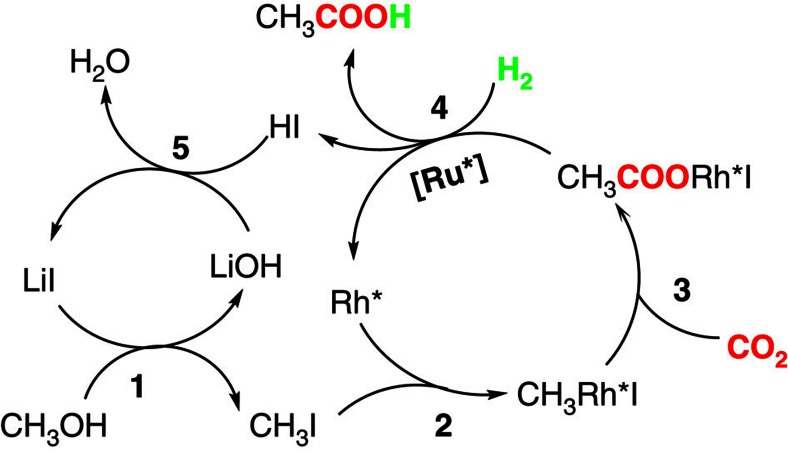
Proposed mechanism. The Ru* and Rh* represent the active Ru and Rh species in the reaction, respectively.

On the basis of all the results above, we proposed the possible mechanism of the reaction (Fig. 6). There are five major steps in the reaction cycle. First, methanol is *in situ* converted into methyl iodide, which is similar to the Monsanto process. (Step 1). It is known that CH_3_I could form spontaneously from methanol and iodine compounds at elevated temperature^32^, which would be promoted by the Lewis acidic cation (Li^+^)^33^. The CH_3_I is a commonly used promoter or intermediate in organic reactions^3^,^8^,^32^,^33^. The tracer experiments using CH_3_OD and CH_3_^18^OH affirmed that the OH broke away from CH_3_OH during the reaction, supporting the formation of CH_3_I (Supplementary Figs 7 and 8). The NMR spectra of the reaction solution using ^13^CH_3_OH as reactant (Supplementary Fig. 10) also verified that the CH_3_ group of methanol is transferred into the acetic acid molecule, which is consistent with the proposed mechanism. Secondly, CH_3_Rh*I was formed via oxidative addition of CH_3_I to the active Rh species (Rh*) (Step 2). The oxidative addition is a basic step in organic synthesis and has been well studied^3^,^32^,^33^. In addition, the tracer experiment and NMR spectra using ^13^CH_3_OH supported the transfer of CH_3_ group during the reaction (Supplementary Figs 9 and 10). The third step was the insertion of CO_2_ into CH_3_Rh*I to form CH_3_COORh*I (Step 3). Rh catalyst was responsible for generating acetic acid (Entry 15 of Table 1). The insertion of CO_2_ into Rh-alkyl bond (including CH_3_–Rh bond) has been well studied^34^, which could be accelerated by enhancing the electron density of the Rh atom. The coordination with imidazole may increase the electron density of the Rh*, which explains the role of imidazole in facilitating the catalytic activity. During the insertion of CO_2_ into the CH_3_–Rh bond, the CH_3_COORh*I formed and the O atom of the C-O adsorbed on the catalyst before further reaction^34^. Next step was reductive elimination of acetic acid from the CH_3_COORh*I in the presence of H_2_, which was promoted by the active Ru species (Ru*) (Step 4). The tracer experiment and NMR spectra using ^13^CH_3_OH indicated that the CH_3_ group of methanol finally entered into the acetic acid molecule (Supplementary Figs 9 and 10). The tracer experiments using CH_3_OD affirmed that the H in the COOH group of acetic acid was from the reactant H_2_ (Supplementary Fig. 7). The catalytic data showed that acetic acid generation was remarkably promoted by the Ru catalyst (Entries 1, 15 of Table 1). The promoting effect of Ru complex on hydrogenating intermediate into product has been reported in other Rh catalysed reactions^35^. Finally, the LiOH and HI generated *in situ* neutralized spontaneously to form LiI and H_2_O (Step 5). At this time, all the catalytic species were regenerated for the next cycle.

In summary, we have developed a route of acetic acid synthesis from methanol, CO_2_ and H_2_. The reaction is efficiently promoted by Ru–Rh bimetallic catalyst. The acetic acid can be generated in large amount at above 180 °C, and the TON of acetic acid exceeds 1,000 after five cycles. The ligand imidazole plays a key role for the high catalytic stability, activity and selectivity of the catalyst. The reaction does not proceed via CO pathway. This route has great potential of application because cheap, easily available starting materials are used and the efficiency is high. Future work is to study the detailed reaction mechanism and design catalytic systems of better performance for industrial application.

## Methods

### Chemicals

Ruthenium carbonyl (Ru_3_(CO)_12_, 98%) and potassium bromide (KBr, 99.9%) were purchased from Adamas Reagent, Ltd. Ruthenium(IV) oxide (RuO_2_, 99.9%, metal basis), Dichlorotris (triphenylphosphine) ruthenium(II) (Ru(PPh_3_)_3_Cl_2_, 97%), Carbonylhydridotris (triphenylphosphine)rhodium(I) (Rh(CO)H_2_(PPh_3_)_3_, Rh>10%), Rhodium(III) chloride hydrate (RhCl_3_·3H_2_O, Rh>38.5%), imidazole (99%), lithium bromide (LiBr, 99%), lithium iodide (LiI, 99.95%), sodium iodide (NaI, 99.5%), potassium iodide, (KI, 99.9%), Tin(IV) iodide (SnI_4_, 99.998%), Triphenylphosphine (PPh_3_, 99%), 2,2′-Bipyridine (99%) and Bis(triphenylphosphoranylidene) ammonium chloride (PPN–Cl, 97%) were obtained from Alfa Aesar China Co., Ltd. Rhodium acetate dimer (Rh_2_(OAc)_4_), lithium chloride (LiCl, 98%) and 1,3-Dimethyl-2-imidazolidinone (DMI, 99%) were purchased from TCI Shanghai Co., Ltd. *N*-Methyl-2-pyrrolidone (NMP, 99.5%), *N*,*N*-dimethylformamide (DMF, 99.5%), cyclohexane (99.5%) and pyridine (99%) were provided by Sinopharm Chemical Reagent Co., Ltd. Methanol (99.5%), tetrahydrofuran (A.R. grade) was obtained from Beijing Chemical Company. Toluene (99.8%, HPLC) was obtained from Xilong Chemical Co., Ltd. Methanol–^13^C (^13^CH_3_OH, 99 atom% ^13^C) and Methanol–^18^O (CH_3_^18^OH, 95 atom% ^18^O) were purchased from Sigma-Aldrich Co. LLC. Methanol–D_1_ (CH_3_OD, 99.5 atom% D) was provided by Beijing InnoChem Science & Technology Co., Ltd. The CO_2_ (99.99%) and H_2_ (99.99%) were purchased Beijing Analytical Instrument Company.

### Catalytic reaction

All the reactions were conducted in a 16 ml Teflon-lined stainless steel batch reactor equipped with a magnetic stirrer. The inner diameter of the reactor was 18 mm. In a typical experiment, known amounts of Ru and/or Rh catalysts, imidazole or another ligand, LiI or another promoter, methanol or (^13^CH_3_OH, CH_3_^18^OH or CH_3_OD if used), and 2 ml DMI or another solvent were loaded sequentially into the reactor. The reactor was purged two times with CO_2_ of 1 MPa in ice-water. At room temperature, CO_2_ in the cylinder was charged into the reactor to desired pressure, and the inlet valve of CO_2_ was closed. Then H_2_ was charged into the reactor until suitable total pressure was reached. The reactor was placed in an air bath of constant temperature, and the magnetic stirrer was started at 800 r.p.m. After reaction, the reactor was cooled in an ice-water bath for 1 h, the residual gas was released slowly and collected in a gasbag. The liquid mixture was analysed by GC (Agilent 7890B) equipped with a flame ionization detector and an HP-5 capillary column (0.32 mm in diameter, 30 m in length) using toluene as the internal standard. Identification of the liquid products was done using a GC–MS (SHIMADZU-QP2010) as well as by comparing the retention times of the standards in the GC traces. The yields of the products were calculated from the GC data. The gaseous samples were analysed using a GC (Agilent 4890D) equipped with a TCD detector and a packed column (Carbon molecular sieve TDX-01, 3 mm in diameter and 1 m in length) using Argon as the carry gas.

### Recycling test

After reaction, the reactor was cooled down using an ice bath and the residual gas was released. The amount of product was determined as discussed above. Then the acetic acid formed and the unreacted methanol in the reactor were removed in a vacuum oven at 85 °C for 5 h. GC analysis confirmed the complete removal of the acetic acid at this condition. The catalytic system (catalyst+promoter+DMI) was used directly for the next run.

## Additional information

**How to cite this article:** Qian, Q. *et al*. Synthesis of acetic acid via methanol hydrocarboxylation with CO_2_ and H_2_. *Nat. Commun.* 7:11481 doi: 10.1038/ncomms11481 (2016).

## Supplementary Material

Supplementary Information Supplementary Figures 1-10

## Figures and Tables

**Figure 1:**
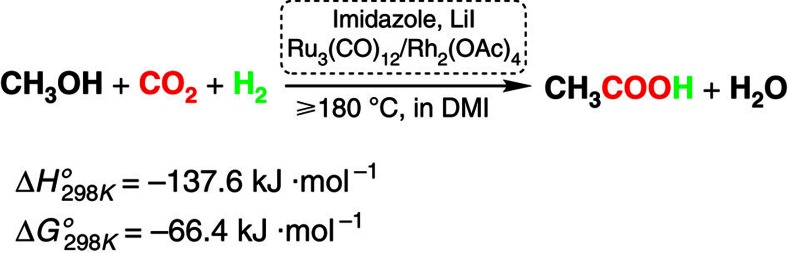
Synthesis of acetic acid by reaction of methanol with CO_2_ and H_2_. In the reaction CO_2_ participates in acetic acid formation with H_2_, and does not via CO.

**Table 1:** Methanol hydrocarboxylation using different catalytic systems.

Entry	Catalyst precursors	Ligand	Promoter	Solvent	TOF* (h^−1^)	Yield† (%)
1	Ru_3_(CO)_12_, Rh_2_(OAc)_4_	Imidazole	LiI	DMI	30.8	70.3
2‡	Ru_3_(CO)_12_, Rh_2_(OAc)_4_	—	LiI	DMI	5.5	12.7
3‡	Ru_3_(CO)_12_, Rh_2_(OAc)_4_	Pyridine	LiI	DMI	3.7	8.3
4‡	Ru_3_(CO)_12_, Rh_2_(OAc)_4_	PPN–Cl	LiI	DMI	12.1	27.7
5‡	Ru_3_(CO)_12_, Rh_2_(OAc)_4_	2,2′-Bipyridine	LiI	DMI	1.1	2.3
6‡	Ru_3_(CO)_12_, Rh_2_(OAc)_4_	PPh_3_	LiI	DMI	0.2	0.3
7‡	Ru_3_(CO)_12_, Rh_2_(OAc)_4_	Imidazole	—	DMI	0	0
8	Ru_3_(CO)_12_, Rh_2_(OAc)_4_	Imidazole	NaI	DMI	2.2	5.0
9	Ru_3_(CO)_12_, Rh_2_(OAc)_4_	Imidazole	KI	DMI	1.1	2.3
10‡	Ru_3_(CO)_12_, Rh_2_(OAc)_4_	Imidazole	SnI_4_	DMI	0	0
11‡	Ru_3_(CO)_12_, Rh_2_(OAc)_4_	Imidazole	LiCl	DMI	0	0
12‡	Ru_3_(CO)_12_, Rh_2_(OAc)_4_	Imidazole	LiBr	DMI	0.4	1.0
13‡	Ru_3_(CO)_12_, Rh_2_(OAc)_4_	Imidazole	KBr	DMI	0	0
14	Ru_3_(CO)_12_	Imidazole	LiI	DMI	0	0
15‡	Rh_2_(OAc)_4_	Imidazole	LiI	DMI	1.9	4.3
16‡	RuO_2_, Rh_2_(OAc)_4_	Imidazole	LiI	DMI	6.1	14.0
17‡	Ru(PPh_3_)_3_Cl_2_, Rh_2_(OAc)_4_	Imidazole	LiI	DMI	0	0
18‡	Ru_3_(CO)_12_, RhCl_3_·3H_2_O	Imidazole	LiI	DMI	0.2	0.3
19‡	Ru_3_(CO)_12_, Rh(CO)H_2_(PPh_3_)_3_	Imidazole	LiI	DMI	0.4	1.0
20	Ru_3_(CO)_12_, Rh_2_(OAc)_4_	Imidazole	LiI	NMP	20.0	45.6
21‡	Ru_3_(CO)_12_, Rh_2_(OAc)_4_	Imidazole	LiI	DMF	0	0
22‡	Ru_3_(CO)_12_, Rh_2_(OAc)_4_	Imidazole	LiI	THF	0	0
23‡	Ru_3_(CO)_12_, Rh_2_(OAc)_4_	Imidazole	LiI	Cyclohexane	0	0
24‡	Ru_3_(CO)_12_, Rh_2_(OAc)_4_	Imidazole	LiI	water	0	0
25‡§	Ru_3_(CO)_12_, Rh_2_(OAc)_4_	Imidazole	LiI	DMI	0	0

TOF, turnover frequency.

Reaction conditions: 40 μmol Ru catalyst and 40 μmol Rh catalyst (based on metals); 0.75 mmol ligand; 3 mmol promoter; 2 ml solvent; 12 mmol MeOH; 4 MPa CO_2_ and 4 MPa H_2_ (at room temperature); 200 °C; and 12 h.

^*^TOF denotes moles of acetic acid produced per mole of Rh catalyst per hour in the steady state.

^†^Yield is based on methanol feedstock (100 × moles of acetic acid product per mole of methanol feedstock).

^‡^Black precipitate was observed after the reaction.

^§^Only H_2_ was used as reactant.

**Table 2:** Effect of reaction parameters on methanol hydrocarboxylation.

Entry	Ru/Rh (μmol)	Imidazole (μmol)	LiI (mmol)	CO_2_/H_2_(MPa)	TOF (h^−1^)	Yield (%)
1	40/40	750	3	1/1	0.1	0.2
2	40/40	750	3	2/2	0.3	0.6
3	40/40	750	3	3/3	4.8	11.0
4	40/40	750	3	4/4	30.8	70.3
5	40/40	750	3	5/5	32.8	75.0
6	40/40	750	3	2/6	1.8	4.0
7	40/40	750	3	6/2	6.3	14.3
8	40/40	750	3	4/0	0	0
9	40/40	750	3	0/4	0	0
10	40/40	450	3	4/4	18.8	43.0
11	40/40	1050	3	4/4	30.4	69.3
12	40/40	750	2	4/4	13.4	30.7
13	40/40	750	4	4/4	20.0	45.6
14	20/60	750	3	4/4	24.5	55.9
15	60/20	750	3	4/4	7.2	16.3
16	20/20	750	3	4/4	9.2	21.0
17	60/60	750	3	4/4	22.5	77.0
18*	40/40	0	3	4/4	0	0
19*	40/40	750	3	4/4	0	0
20†	40/40	0	3	4/4	0	0
21†	40/40	750	3	4/4	0	0

TOF, turnover frequency.

Reaction conditions: Ru_3_(CO)_12_/Rh_2_(OAc)_4_ were used as catalysts and their dosage was based on metal; imidazole was used as ligand; LiI was used as promoter; 12 mmol MeOH; 2 ml DMI; 200 °C; and 12 h.

^*^CO and H_2_ were used as reactants.

^†^CO_2_ and H_2_ were used as reactants.
