# Supplementary material for: Synthesis of acetic acid via methanol hydrocarboxylation with CO2 and H2
Source: Nat Commun. 2016 May 11;7:11481. doi: 10.1038/ncomms11481 (PMC4865843; doi:10.1038/ncomms11481)
Supplement: Supplementary Information — Supplementary Figures 1-10 [file ncomms11481-s1.pdf]

## Supplementary Figures

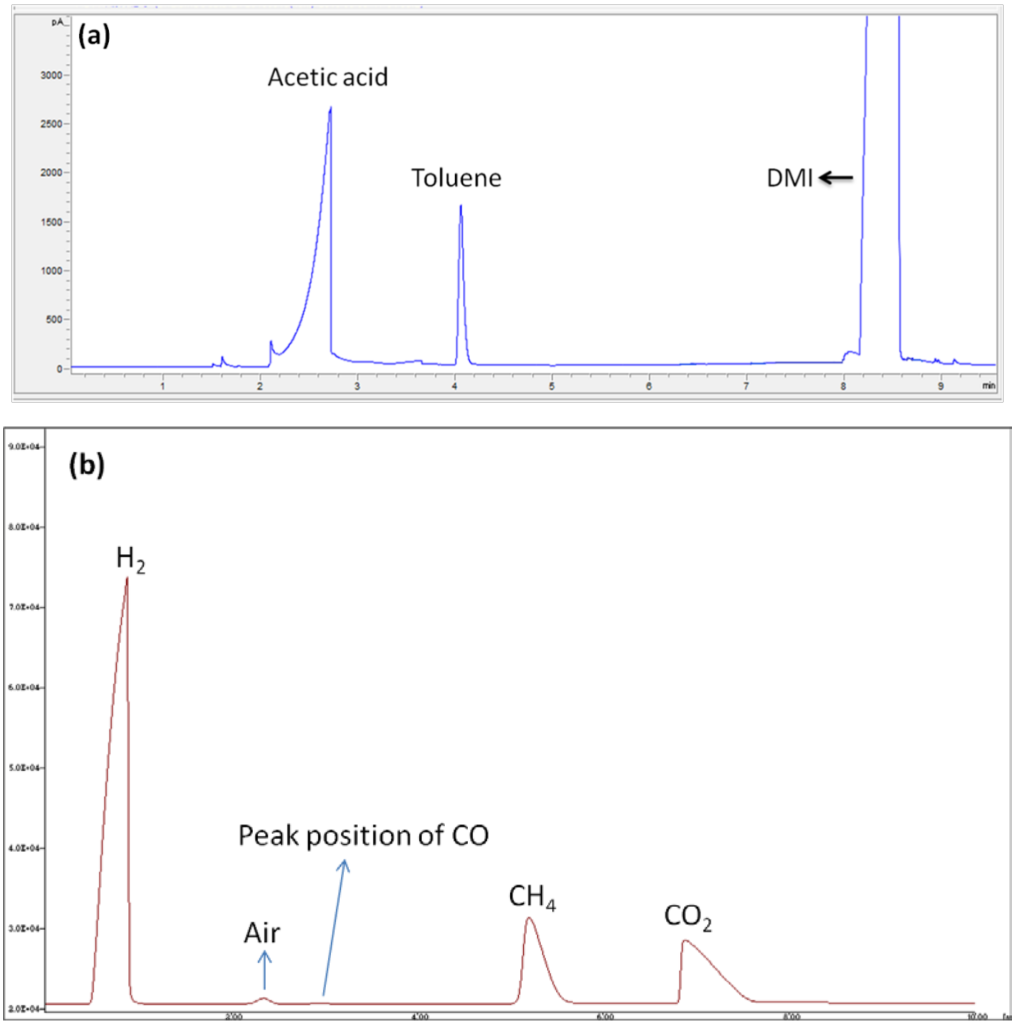


**Supplementary Fig. 1. The GC traces of the products of methanol hydrocarboxylation.** *(a)* liquid sample (toluene as internal standard), *(b)* gaseous sample. Condition: 40 μmol $Ru_3(CO)_{12}$ and 40 μmol $Rh_2(OAc)_4$ (based on metals), 0.75 mmol imidazole, 3 mmol LiI, 2 mL DMI, 12 mmol MeOH, 4 MPa $CO_2$ and 4 MPa $H_2$ (at room temperature), 200 $^{o}$C, and 12 h.

Notes:

(1) The determined mole response factors of $H_2$, CO and $CH_4$ are 6.32, 0.97 and 3.51 times of that of $CO_2$.

(2) The weak air peaks was caused by sampling and injecting operation.

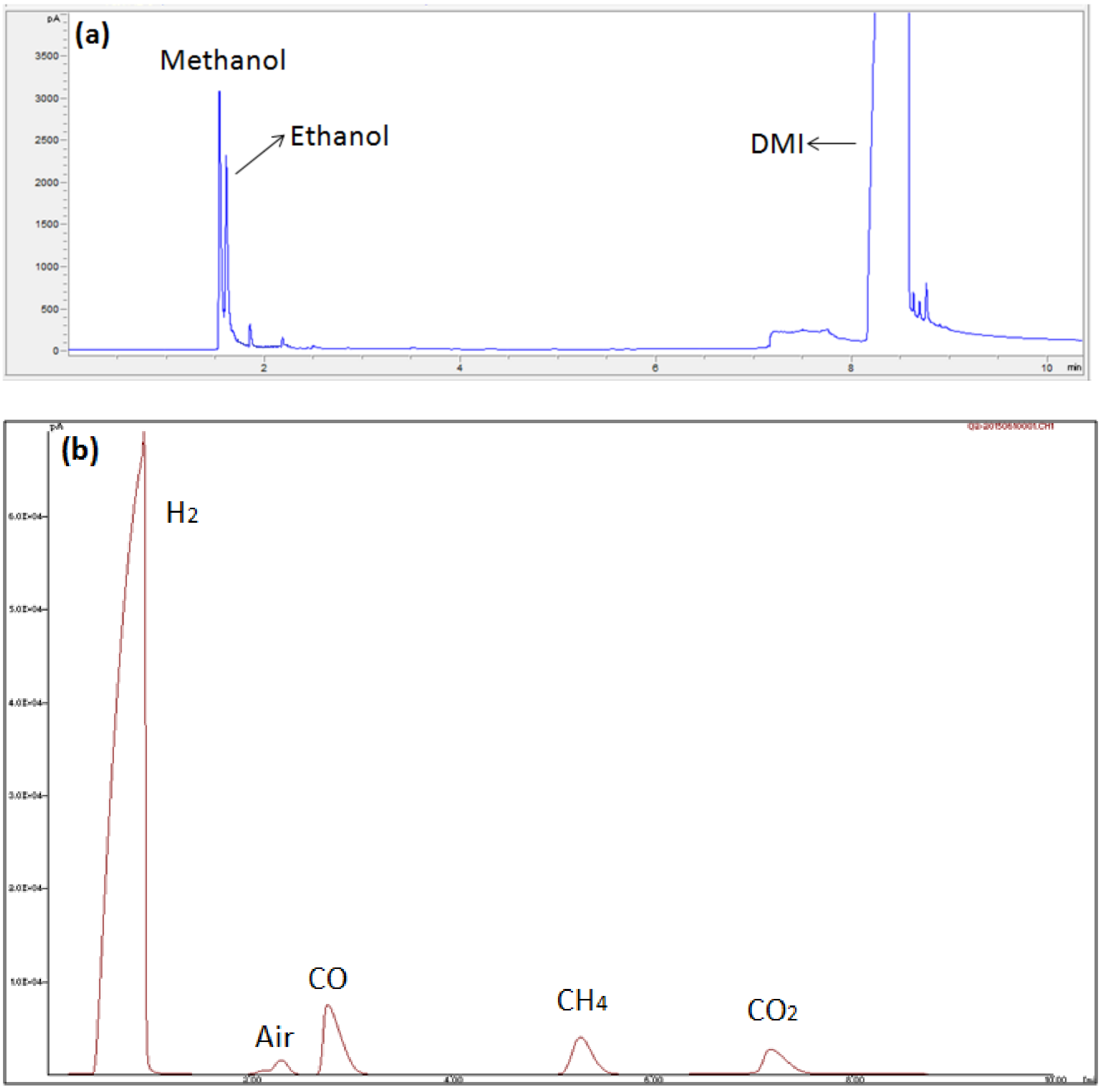


**Supplementary Fig. 2. The GC traces of the products of CO hydrogenation without imidazole.** *(a)* liquid sample, *(b)* gaseous sample. Condition: 40 μmol $Ru_3(CO)_{12}$ and 40 μmol $Rh_2(OAc)_4$ (based on metals), 3 mmol LiI, 2 mL DMI, 4 MPa CO and 4 MPa $H_2$ (at room temperature), 200 $^{o}$C, and 12 h.

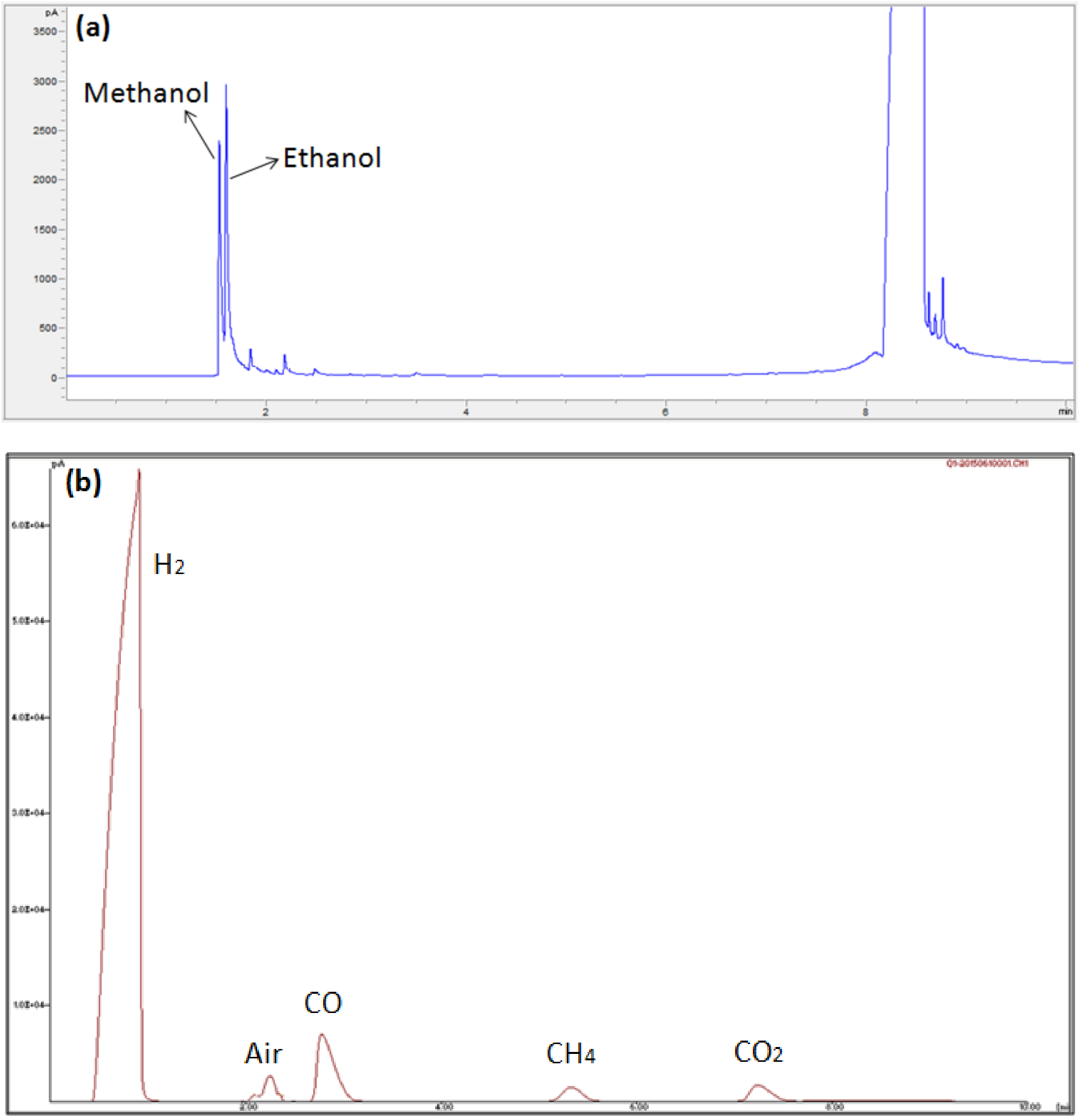


**Supplementary Fig. 3. The GC traces of the products of CO hydrogenation with imidazole.** *(a)* liquid sample, *(b)* gaseous sample. Condition: 40 μmol $Ru_3(CO)_{12}$ and 40 μmol $Rh_2(OAc)_4$ (based on metals), 0.75 mmol imidazole, 3 mmol LiI, 2 mL DMI, 4 MPa CO and 4 MPa $H_2$ (at room temperature), 200 $^{o}$C, and 12 h.

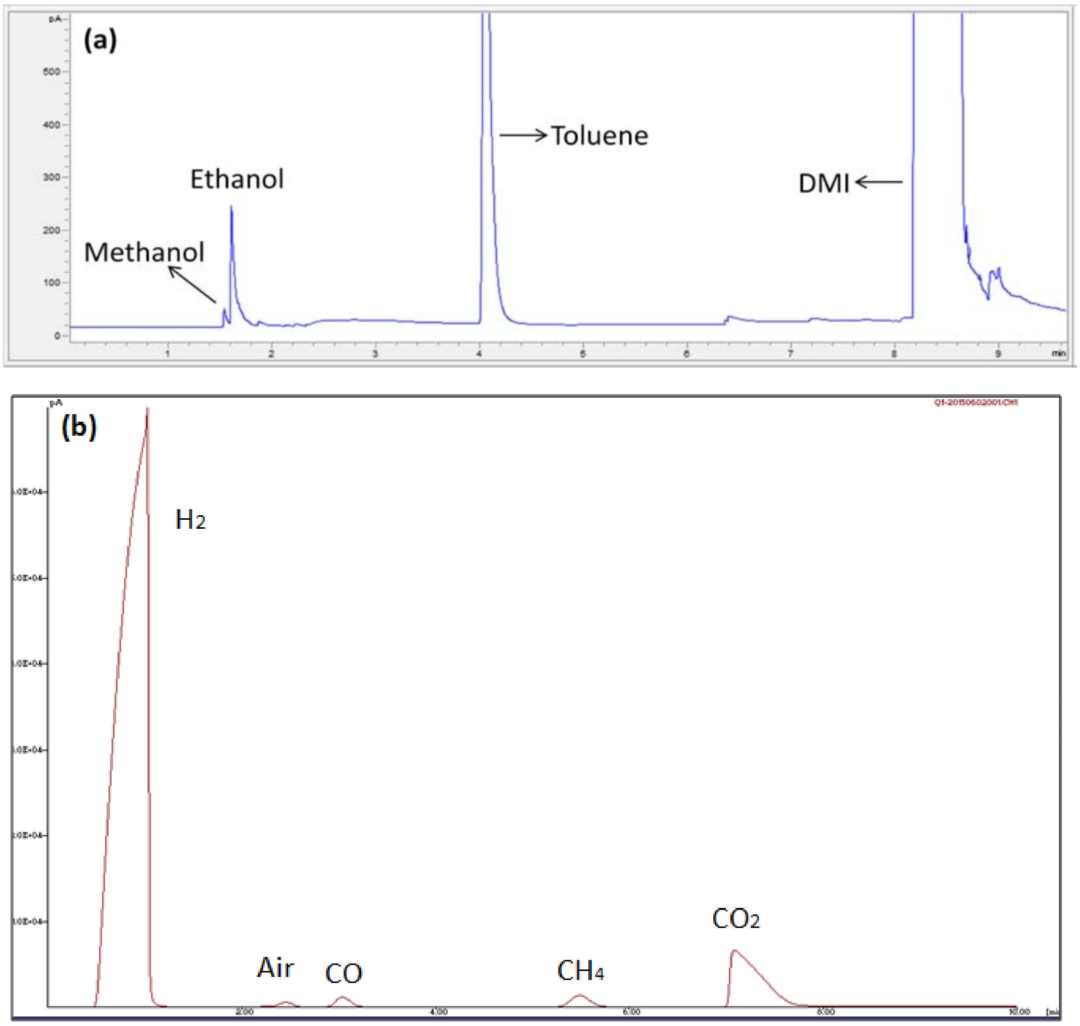


**Supplementary Fig. 4. The GC traces of the products of $CO_2$ hydrogenation without imidazole.** *(a)* liquid sample (toluene as internal standard), *(b)* gaseous sample. Condition: 40 μmol $Ru_3(CO)_{12}$ and 40 μmol $Rh_2(OAc)_4$ (based on metals), 3 mmol LiI, 2 mL DMI, 4 MPa $CO_2$ and 4 MPa $H_2$ (at room temperature), 200 $^{o}$C, and 12 h.

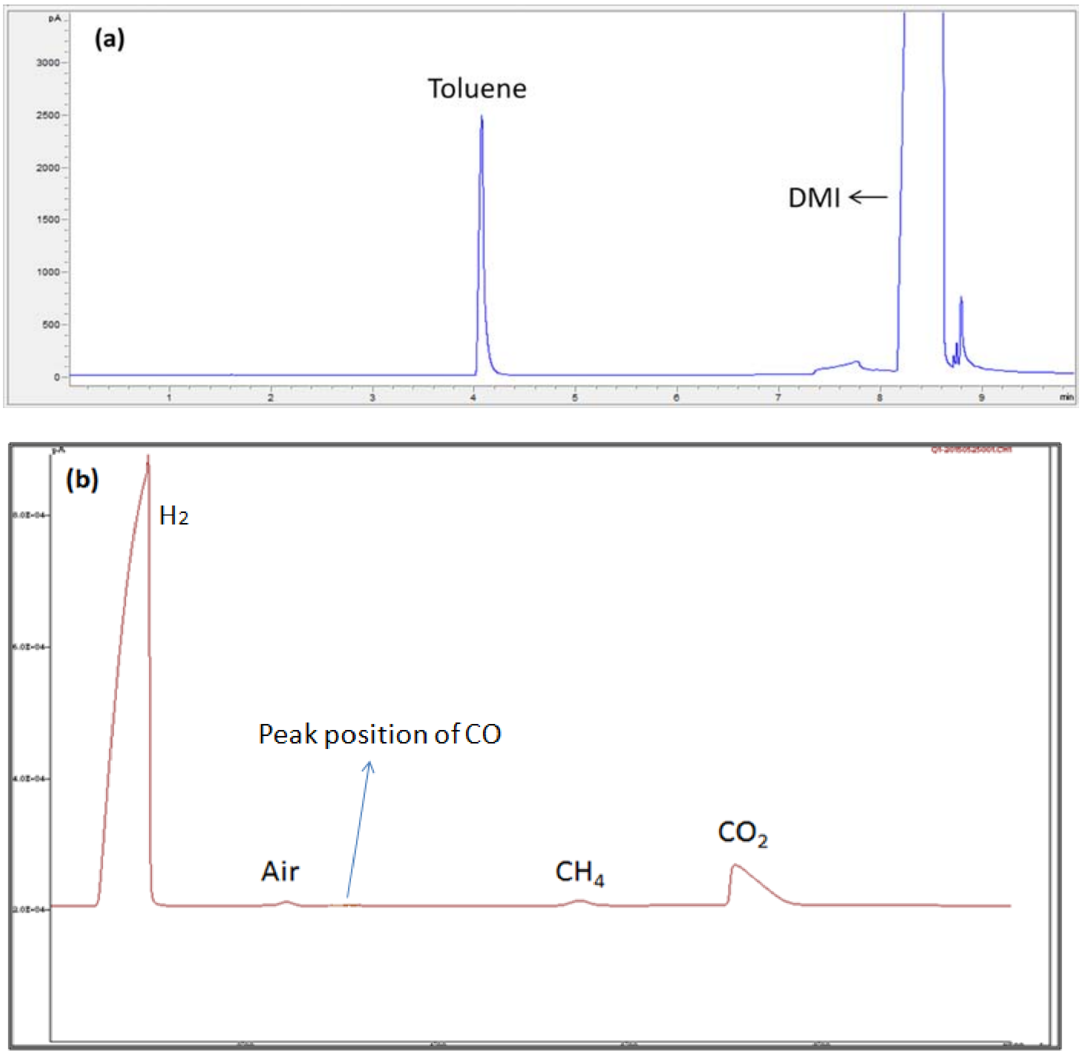


**Supplementary Fig. 5. The GC traces of the products of $CO_2$ hydrogenation with imidazole.** *(a)* liquid sample (toluene as internal standard), *(b)* gaseous sample. Condition: 40 μmol $Ru_3(CO)_{12}$ and 40 μmol $Rh_2(OAc)_4$ (based on metals), 0.75 mmol imidazole, 3 mmol LiI, 2 mL DMI, 4 MPa $CO_2$ and 4 MPa $H_2$ (at room temperature), 200 $^{o}$C, and 12 h.

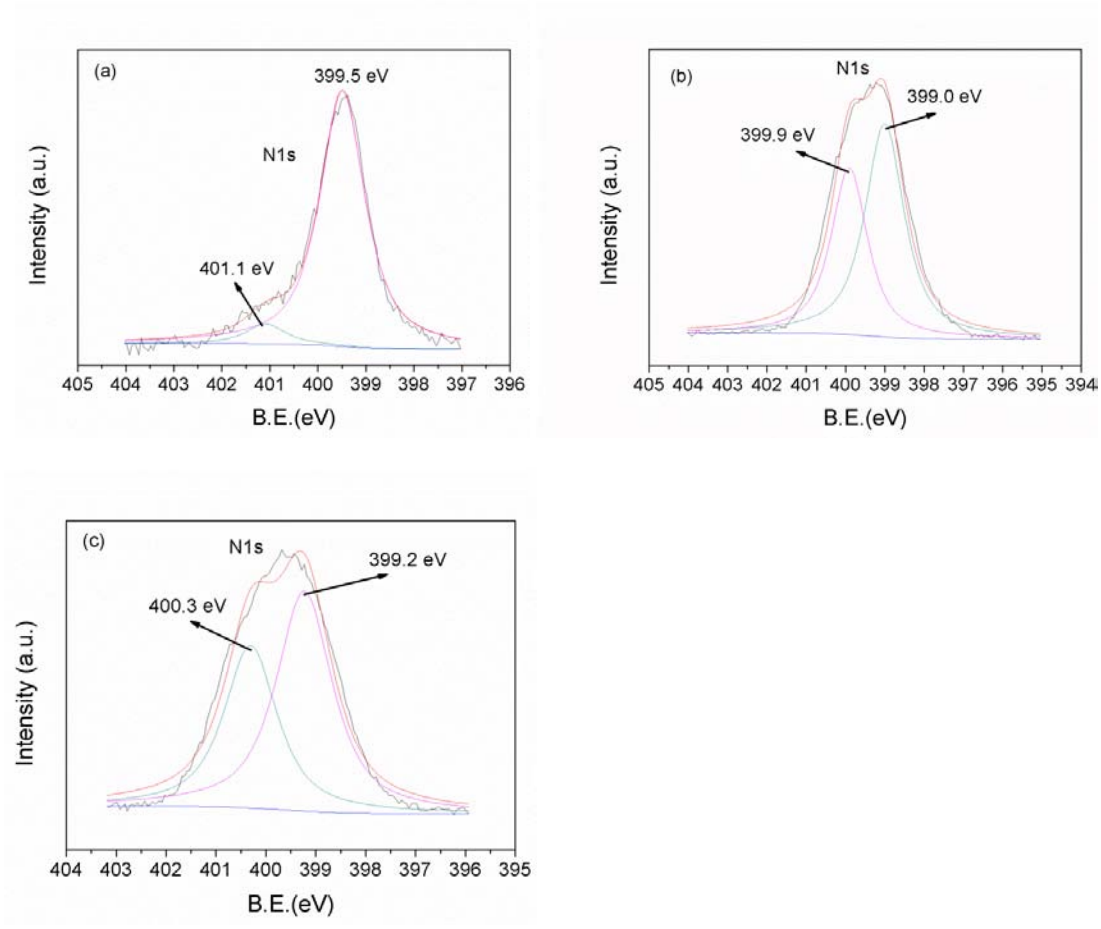


**Supplementary Fig. 6. The results of XPS analysis.** *(a)* imidazole, *(b)* imidazole-$Ru_3(CO)_{12}$ compound, *(c)* imidazole-$Rh_2(OAc)_4$ compound.

Notes: X-ray photoelectron spectroscopy (XPS) data were obtained with an ESCALab220i-XL electron spectrometer from VG Scientific using 300W AlKα radiation. The base pressure was about $3\times10^{-9}$ mbar. The binding energies were referenced to the C1s line at 284.8 eV from adventitious carbon.

The preparation of imidazole-$Ru_3(CO)_{12}$ compound was conducted at room temperature. In the experiment, 0.0085 g $Ru_3(CO)_{12}$ (40 μmol Ru) was dissolved in 20 mL dioxane, 0.05 g imidazole (750 μmol) was dissolved in 20 mL methanol. The above solutions were mixed and stirred for 5 h, then 40 mL diethyl ether was added to precipitate the target compound. The compound was washed with additional 40 mL diethyl ether for 2 times and dried in vacuum before the XPS analysis.

The preparation of imidazole-$Rh_2(OAc)_4$ compound was conducted at room temperature. In the experiment, 0.0088 g $Rh_2(OAc)_4$ (40 μmol Rh) and 0.05 g imidazole (750 μmol) was dissolved in 20 mL methanol respectively. The above solutions were mixed and stirred for 5 h, and then 40 mL diethyl ether was added to precipitate the target compound. The compound was washed with additional 40 mL diethyl ether for 2 times and dried in vacuum before the XPS analysis.

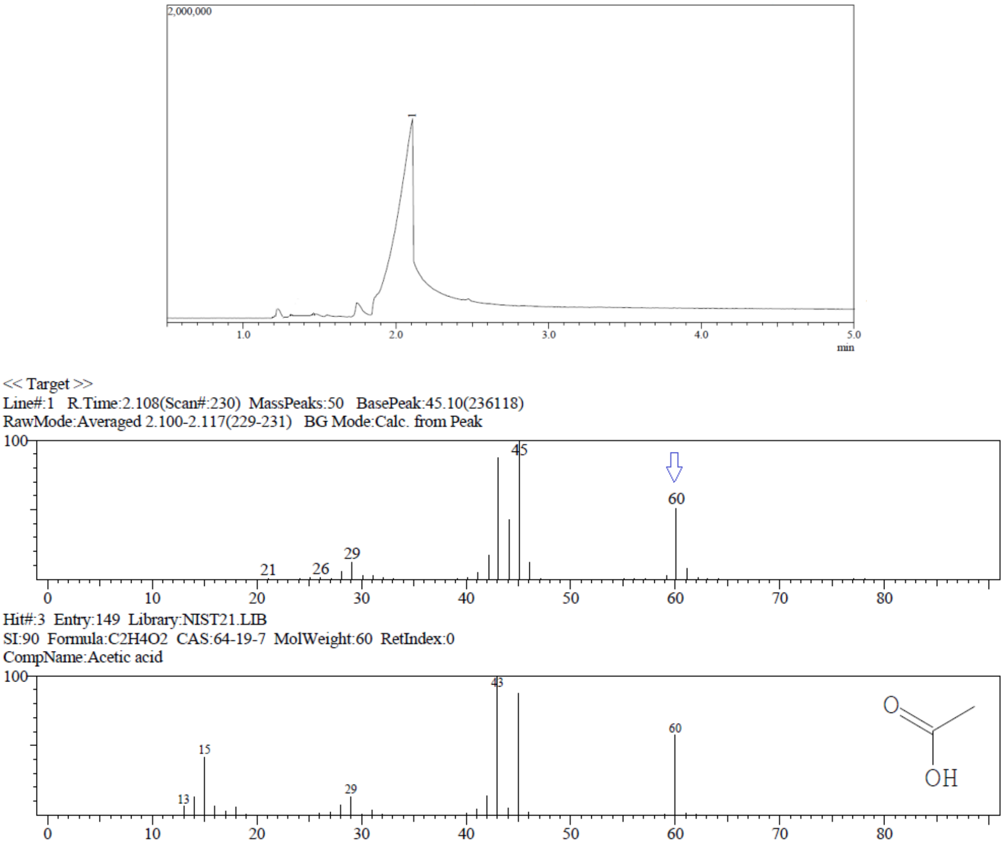


**Supplementary Fig. 7. The GC-MS spectra of reaction solution using $CH_3OD$ instead of methanol.** Condition: 40 μmol $Ru_3(CO)_{12}$ and 40 μmol $Rh_2(OAc)_4$ (based on metals), 0.75 mmol imidazole, 3 mmol LiI, 2 mL DMI, 12 mmol $CH_3OD$, 4 MPa $CO_2$ and 4 MPa $H_2$ (at room temperature), 200 $^oC$, and 12 h.

Note: The molecular weight of acetic acid generated in the reaction was still 60 Daltons. This result supports three deductions.

1. The $CH_3$ and OD group broke away during the reaction. Otherwise, the molecular weight of acetic acid should be 61 Daltons.

2. The $CO_2$ directly participated in the reaction. If methanol carbonylation with CO dominated in the reaction, the OD group generated in situ would take part in the formation of acetic acid with the $CH_3CORh^*I$ intermediate and the molecular weight of acetic acid should be 61 Daltons. The mechanism of rhodium catalyzed methanol carbonylation was reported elsewhere (*Ref 3*).

3. H atom in the COOH group of acetic acid was from the reactant $H_2$. Otherwise, the molecular weight of acetic acid should be 61 Daltons.

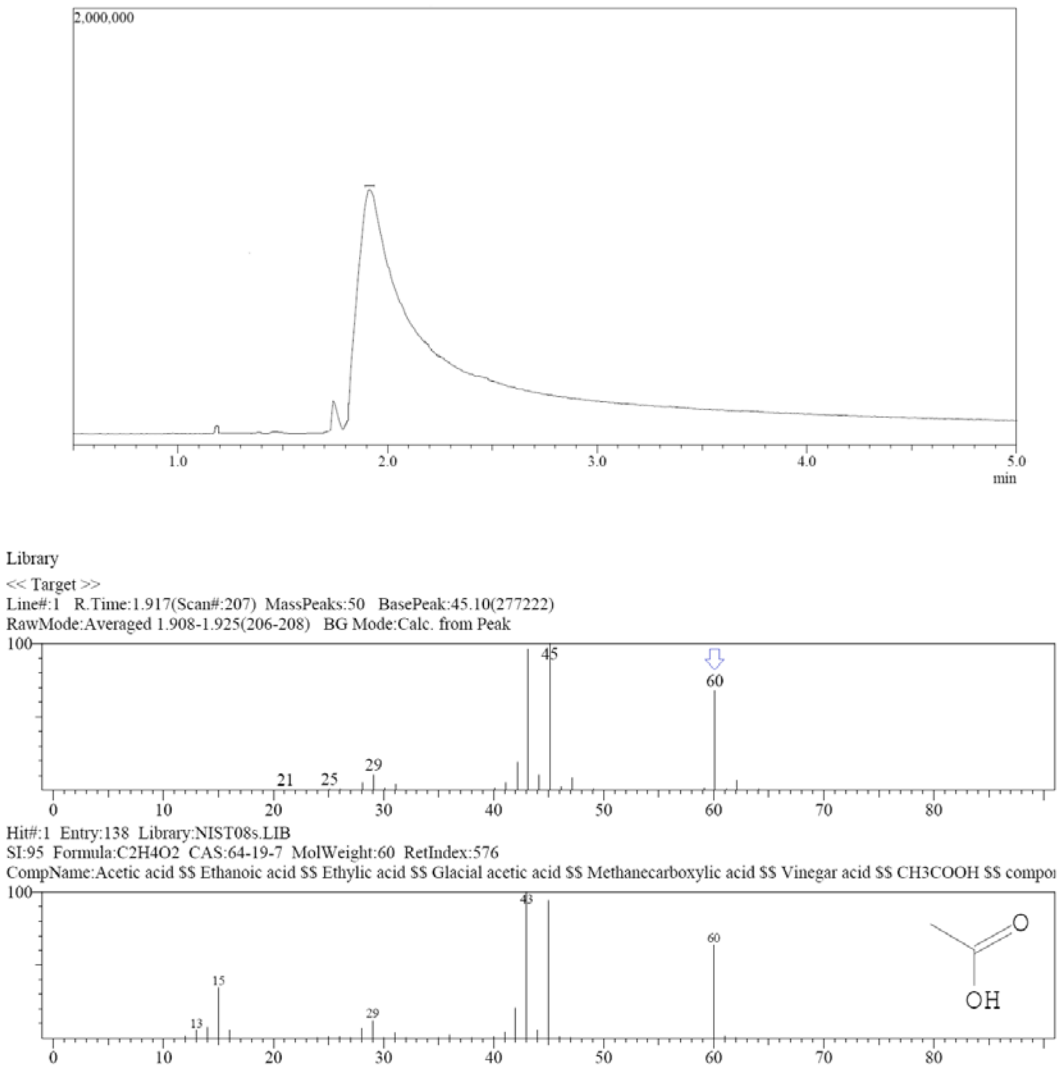


**Supplementary Fig. 8. The GC-MS spectra of reaction solution using $CH_3{}^{18}OH$ instead of methanol.** Condition: 40 μmol $Ru_3(CO)_{12}$ and 40 μmol $Rh_2(OAc)_4$ (based on metals), 0.75 mmol imidazole, 3 mmol LiI, 2 mL DMI, 12 mmol $CH_3{}^{18}OH$, 4 MPa $CO_2$ and 4 MPa $H_2$ (at room temperature), 200 $^{o}$C, and 12 h.

Note: The molecular weight of acetic acid synthesized was still 60 Daltons. This result supports two deductions.

1. The $CH_3$ and $^{18}OH$ group broke away during the reaction. Otherwise, the molecular weight of acetic acid should be 62 Daltons.

2. The $CO_2$ directly participated in the reaction. If methanol carbonylation with CO dominated in the reaction, the $^{18}OH$ group generated in situ would take part in the formation of acetic acid with the $CH_3CORh^*I$ intermediate and the molecular weight of acetic acid should be 62 Daltons. The mechanism of rhodium catalyzed methanol carbonylation was reported elsewhere (*Ref 3*).

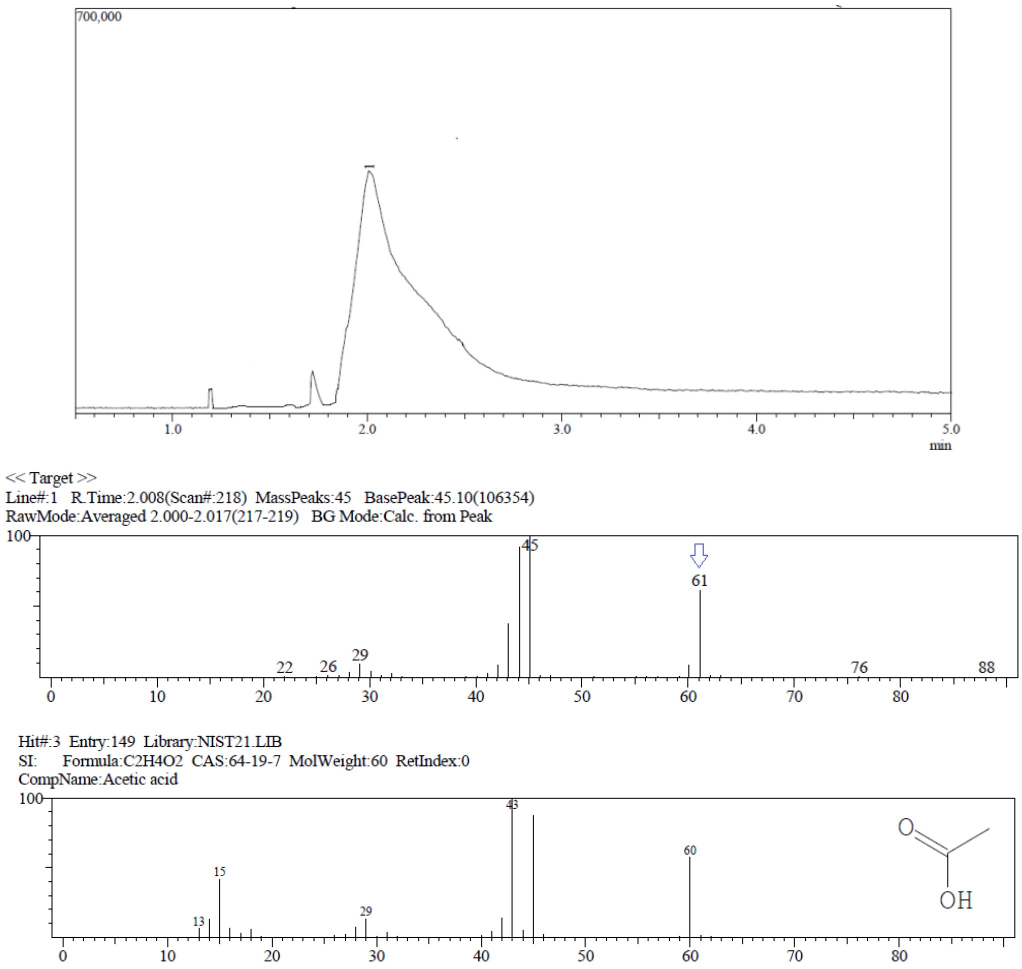


**Supplementary Fig. 9. The GC-MS spectra of reaction solution using $^{13}CH_3OH$ instead of methanol.** Condition: 40 μmol $Ru_3(CO)_{12}$ and 40 μmol $Rh_2(OAc)_4$ (based on metals), 0.75 mmol imidazole, 3 mmol LiI, 2 mL DMI, 12 mmol $^{13}CH_3OH$, 4 MPa $CO_2$ and 4 MPa $H_2$ (at room temperature), 200 °C, and 12 h.

Note: The molecular weight of acetic acid formed in the reaction was 61 Daltons. This demonstrates that the two C atoms in the acetic acid product were from $^{13}C$ of $^{13}CH_3OH$ and C of $CO_2$ respectively.

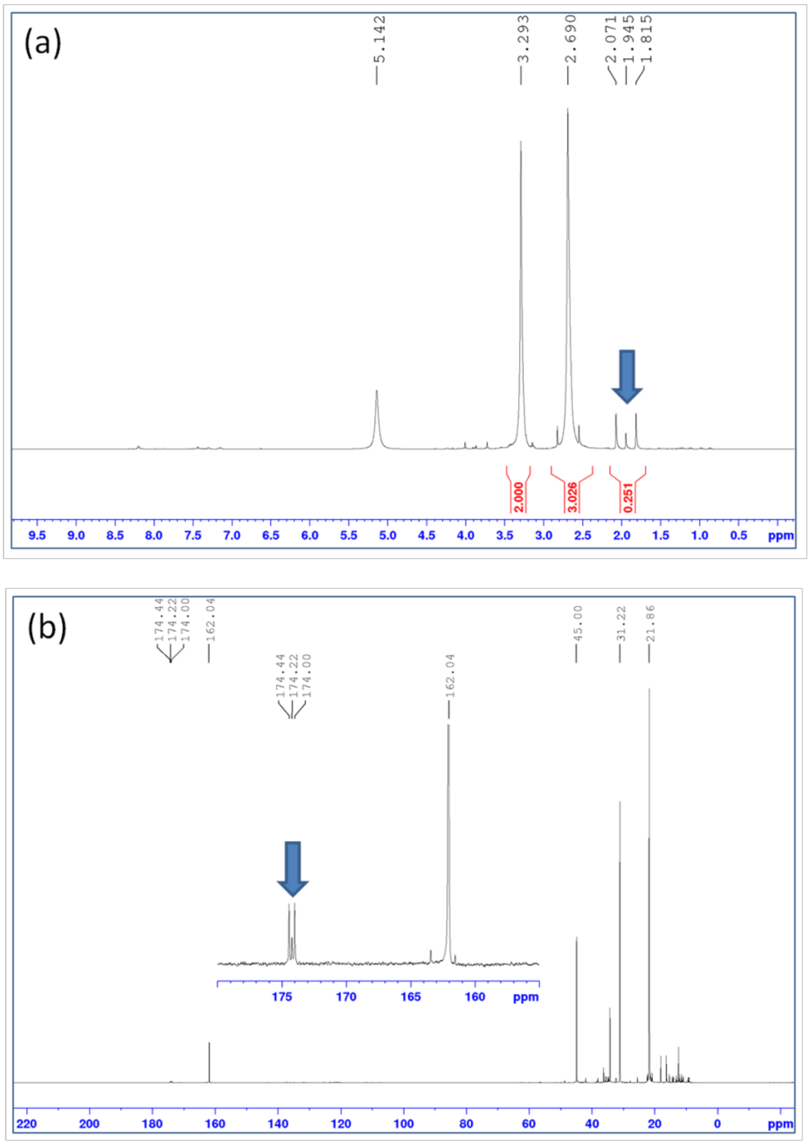


**Supplementary Fig. 10. The NMR spectra of reaction solution using $^{13}CH_3OH$ instead of methanol.** *(a)* $^1H$ NMR, *(b)* $^{13}C$ NMR. Condition: 40 μmol $Ru_3(CO)_{12}$ and 40 μmol $Rh_2(OAc)_4$ (based on metals), 0.75 mmol imidazole, 3 mmol LiI, 2 mL DMI, 12 mmol $^{13}CH_3OH$, 4 MPa $CO_2$ and 4 MPa $H_2$ (at room temperature), 200 °C, and 12 h.

Note: In the $^1H$ NMR spectrum, the proton signal of $^{13}CH_3$ group on the acetic acid molecule splits into two peaks by the coupling with $^{13}C$ atom. In the $^{13}C$ NMR spectrum, the signal of carbonyl group became weaker and splits into dual peaks, which is caused by the coupling with the adjacent $^{13}C$ atom in the $^{13}CH_3$ group. Both $^1H$ NMR and $^{13}C$ NMR spectra confirmed that the $CH_3$ group in acetic acid molecule is from methanol, *i.e.*, $CH_3$ group of $CH_3OH$ is transferred into the acetic acid product in the reaction.
